# Social Support and Adherence to Self-Care Behavior Among Patients With Coronary Heart Disease and Heart Failure: A Systematic Review

**DOI:** 10.5964/ejop.12131

**Published:** 2024-02-29

**Authors:** Athira Babygeetha, Dhanalakshmi Devineni

**Affiliations:** 1 Department of Applied Psychology, Pondicherry University, Puducherry, India; Università Degli Studi dell'Aquila, L'Aquila, Italy

**Keywords:** social support, adherence to self-care, coronary heart disease, heart failure, systematic review

## Abstract

Cardiovascular diseases stand out as the foremost cause of mortality on a global scale and encompass conditions that require long term self-care. Coronary heart disease and heart failure are two cardiovascular conditions that require significant lifestyle modifications. Adherence to self-care is a multifaceted phenomenon, and is influenced by various factors that include social, economic, disease-related and healthcare system-related factors. A key factor in adherence to self-care in chronic illnesses is social support. To explore this relationship between social support and adherence to self-care, a systematic review was carried out across Scopus, EBSCO host and ProQuest from October 2022 to February 2023 using predefined search criteria. Studies from inception to February 2023 were considered for the review, ultimately incorporating a total of 11 studies. Six studies had an adult population with coronary heart disease while the remaining five had adults with heart failure. All the studies reported a significant positive correlation between social support and adherence to self-care. Our findings revealed that social support plays a significant role in promoting self-care, emphasizing the need for a holistic understanding of self-care to develop effective interventions. Along with self-report measures, objective measures should be used to assess adherence accurately. There is a need for scales that assess all aspects of self-care, as well as the development of new interventions and teaching strategies to facilitate the individual’s self-care journey. In addition, family members and trusted resources should be involved in encouraging self-care, and interventions should target both patients and their family members.

*Chronic disease*s persistently affect the well-being and quality of life, and their prevalence is increasing at an alarming pace in both developed and developing countries (Sprangers et al., 2000). Among the various chronic diseases, cardiovascular diseases (CVDs) constitute the foremost cause of death globally (World Health Organization [WHO], 2021). The incidence of cardiovascular disease has surged from approximately 285 million in 1990 to 523 million in 2019 (World Heart Federation, n.d.). Furthermore, the mortality rate has steadily increased, from 12.1 million in 1990 to approximately 18.6 million in 2019 (Roth et al., 2020). According to the World Heart Federation (n.d.), the prevalence of coronary heart disease (CHD), rheumatic heart disease, and stroke has increased exponentially from 1990 to 2019, with the highest death rate observed for coronary/ischemic heart disease (IHD).

CVDs encompass various conditions requiring long-term self-care. Compared to other CVDs, coronary heart disease, also called coronary artery disease (CAD) or ischemic heart disease (IHD), and heart failure require greater lifestyle modifications (Wenn et al., 2022). According to the Mayo Clinic (2022) and the Centers for Disease Control and Prevention (2021), coronary artery disease can lead to symptoms such as chest pain (angina), heart attack (myocardial infarction), and eventually heart failure. An analysis of the epidemiological burden of heart failure indicates that it is primarily caused by ischemic or coronary heart disease (43% of all cases), followed by other conditions (Lippi & Sanchis-Gomar, 2020). Heart failure is associated with significant clinical and economic burdens, posing challenges for healthcare systems and providers, as approximately 17–45% of patients die within a year of hospitalization, and most die within five years of admission (Ponikowski et al., 2014).

The Heart Failure Association of the European Society of Cardiology has identified appropriate self-care and lifestyle changes as significant factors that can help manage the condition without worsening it (McDonagh et al., 2021). Similarly, the American Heart Association (2015) posited that appropriate self-care and a healthy lifestyle can considerably delay the progression of CHD (Iestra et al., 2005) and positively impact the long-term prognosis of the condition. Hence, focused attention on self-care and the adoption of a healthy lifestyle can significantly reduce the burden of CVD by addressing the specific needs of CHD and heart failure.

Adherence to self-care activities is essential for the prevention and management of CVDs (Riegel et al., 2017). The World Health Organization (WHO, 2003) defines adherence as “the extent to which a person's behavior corresponds with the agreed recommendations of a healthcare provider.” To reduce the risk of recurrent events, secondary prevention of CVD involves adherence to treatment regimens and lifestyle recommendations (Fuster, 2014; National Heart Foundation of Australia, 2011). Poor adherence is associated with increased hospitalization and premature mortality rates among patients with heart failure and CHD (Kumbhani et al., 2013; Mozaffarian et al., 2016).

Self-care is a multi-faceted and complex phenomenon that requires knowledge, skills, and lifelong commitment (Horowitz et al., 2004), and patients spend most of their time outside of clinical settings engaging in self-care behaviors (Riegel et al., 2017). Despite its association with improved symptom control and reduced hospitalization (Attebring et al., 2005), non-adherence to recommended treatment strategies is prevalent among individuals with CVD in developed countries, with approximately 50% failing to adhere (Laba et al., 2013). The theory of self-care for chronic illness provides a comprehensive picture of self-care and posits that it involves decision-making for maintaining and managing one's health and is comprised of three core elements: self-care maintenance, self-care management, and self-care monitoring (Riegel et al., 2012). This approach to self-care provides a more comprehensive picture than viewing self-care as mere activities or behaviours.

Adherence to self-care in chronic illnesses such as CVD is influenced by various factors, including social, economic, disease-related, and healthcare system-related factors (Schafer, 2017, as cited in Gast & Mathes, 2019; WHO, 2003). The role of social support in influencing health behaviors has been extensively researched, and patients with CVD often require assistance from family and friends to initiate and maintain self-care practices (Graven & Grant, 2014; Riegel et al., 2007). A systematic review by Rashidi et al. (2020) demonstrated that social support contributes significantly to the adherence to treatment plans in chronic diseases. Conversely, the lack of social support has been linked to a higher likelihood of high-risk factors, such as atherosclerosis, myocardial infarction, slower recovery from CVD events, and increased mortality (Frasure-Smith et al., 2000; Lett et al., 2009; Rosengren et al., 2004; Rozanski et al., 1999).

Social support is a complex construct that encompasses an individual's social relationships and the specific functions they serve (Uchino, 2004). The functions of social networks can be categorized into received and perceived support, encapsulating emotional, instrumental, and informational support (Luszczynska et al., 2013; Rashidi et al., 2020; Uchino, 2004). Emotional support involves expressions of care and concern, instrumental support involves material aid or fulfilment of tangible needs, and informational support refers to providing information and guidance (Schultz et al., 2022). Additionally, appraisal and affirmative support are closely related to emotional support (Berkman & Glass, 2000; Langford et al., 1997).

According to the Health Action Process Approach (HAPA, Schwarzer, 2016), social support is an important volitional factor and significantly promotes adaptive cognitive appraisal processes and coping efforts during difficult times (Reyes-Fernández et al., 2014). Regarding health, social support helps individuals adopt and maintain healthy lifestyle changes (Teleki et al., 2022), which is crucial for long-term prognosis. The situation-specific theory of self-care (Riegel & Dickson, 2008), views it as a process shaped by an individual's health beliefs and social factors, including social support (Artinian et al., 2002), and is specific to the situation and context in which it occurs.

Thus, research in chronic illnesses has revealed that social support has a significant influence on adherence to self-care. The results have, however, been inconsistent and inconclusive regarding chronic conditions such as CHD and heart failure. In this systematic review, the authors are investigating how past studies have highlighted the relationship between social support and adherence to self-care in these conditions. To the best of our knowledge, no systematic review of cross-sectional studies has been conducted to explore the association between these two variables for patients with CHD and heart failure. Hence, this study aims to gather prospective evidence on how the two variables are related, providing new insights that can be translated into practice.

## Method

The systematic review was performed as per the guidelines given in Preferred Reporting Items for Systematic Review and Meta-Analyses (PRISMA, Page et al., 2021). Initially, a scoping search was conducted between October 2022 and January 2023 to identify relevant studies exploring the association between social support and adherence to self-care among patients with CHD and heart failure. The search was conducted on multiple databases, including Scopus, EBSCOhost, ProQuest, Google Scholar, and PROSPERO using the search terms “social support,” “adherence to self-care behavior,” “coronary artery disease,” “heart failure,” “cardiovascular diseases” and “systematic review.” However, the search results did not identify any existing or ongoing systematic reviews exploring the correlation between the proposed study variables among individuals with CHD and heart failure.

### Eligibility Criteria

The inclusion criteria for the eligible studies were as follows: (1) peer-reviewed studies in English, (2) studies including patients with CHD and/or heart failure, (3) quantitative studies including other chronic conditions but with a separate analysis conducted for CHD and/or heart failure, (4) studies undertaking the quantitative measurement of the relationship between social support and adherence to self-care behaviors, with social support including different forms of structural and functional support, such as emotional and/or belonging support, informational support, and instrumental and/or tangible support. Adherence to self-care behavior included adherence to medication; physical activity; diet; weight control; cessation of smoking; and reduced alcohol use (Riegel et al., 2017), (5) Studies conceptualizing self-care from the perspective of the Middle range theory of chronic illness (Riegel et al., 2012), which defines self-care in chronic illness as comprising self-care maintenance, management, and monitoring, (6) multivariate studies that computed Pearson’s/Spearman’s correlation between the relevant variables, and (7) review papers (systematic reviews, scoping reviews, meta-analyses) that contained studies relevant to the variables in the current review.

The exclusion criteria included: (1) randomized controlled trials (RCTs), and intervention, longitudinal, cohort, and qualitative studies, (2) multivariate studies that have not computed the correlation between the study variables separately/apart from the multivariate analyses, (3) studies with insufficient measurement of variables, (4) studies focusing on the association between social support and self-care adherence in the emergency department of hospitals, (5) studies assessing dyadic processes between patients and caregivers, where social support was only considered from the caregiver’s perspective, (6) studies focused on tool development, (7) studies including patients with other comorbid chronic diseases (diabetes, chronic renal disease, cancer, and diagnosis of severe mental disorders such as depression, anxiety disorders, and schizophrenia), (8) conference papers, (9) studies published in languages other than English, and (10) editorials, opinion papers/commentaries, and conceptual papers.

### Information Sources

The EBSCO, Scopus and ProQuest databases were searched from inception up to February 2023. The search was performed on February 7 and 12, 2023. Relevant studies were also identified from the references of the included studies and Google searches. The authors of these studies were contacted when further information was required.

### Search Strategy

The search strategy was formulated based on database-specific headings, Medical Subject Headings (MeSH), and relevant terms were identified through free-text search. The terms used included synonyms for “adherence to self-care behavior,” “coronary heart disease,” “heart failure” and “social support.” All terms were combined using Boolean logic commands appropriate for each database.

### Study Selection

Two reviewers independently assessed the article titles and abstracts retrieved from the search databases and additional sources mentioned. Subsequently, based on the predefined criteria they read the full text of the articles. Data were extracted using Microsoft Excel. Any discrepancies between the reviewers were resolved through discussions, and a consensus was reached.

### Quality Assessment

The quality assessment of the selected studies was conducted using the Joanna Briggs Institute checklist (Moola et al., 2020). Each reviewer independently evaluated the included studies, and discrepancies were resolved through discussion. The quality appraisal aimed to determine the methodological strengths and weaknesses of the selected studies, rather than excluding them based on quality.

### Data Extraction and Synthesis

The included studies were reviewed based on various aspects, including author, publication year, population characteristics (number and demographics of the study group), study setting, variables measured, instruments used, and study outcomes. A narrative synthesis approach was employed to synthesize the findings of the included studies, as meta-analyses were deemed unsuitable owing to the heterogeneity among the selected studies.

## Results

### Study Selection and Characteristics of the Studies

**Figure 1:**
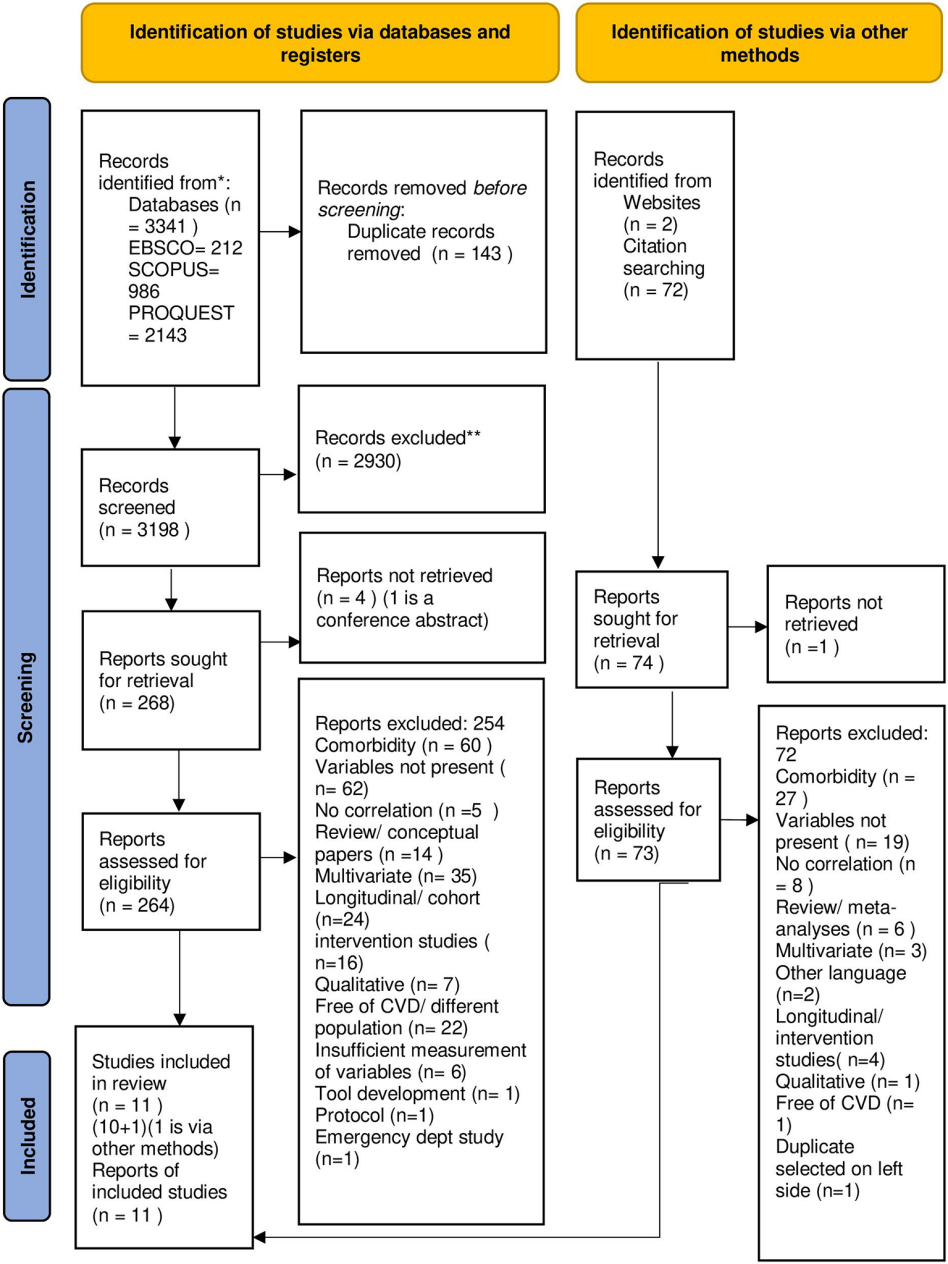
Flowchart of the Included Studies

The search process identified 3341 papers, with 212 from EBSCOhost, 986 from Scopus, and 2143 from ProQuest. In addition, 74 papers were identified from the references of the selected studies and Google searches. After removing duplicates, 3198 studies were considered for the title and abstract screening. Out of these, 2930 studies were deemed irrelevant to the review question and were excluded, resulting in 268 studies for full paper retrieval. However, full papers were not available for three of the studies despite contacting the authors. Finally, 264 papers were filtered for full-text reading and only 11 studies were selected based on the predefined inclusion and exclusion criteria. One of the 11 studies was selected using an alternative method (Google Scholar search). Figure 1 presents an illustration of the study selection process.

Nine of the selected studies employed a cross-sectional design (Fang et al., 2017; Ginting et al., 2016; Hammash et al., 2017; Khaledi et al., 2014; Mei et al., 2019; Rocha et al., 2017; Rokhmah et al., 2020; Tawalbeh et al., 2015; Yunus & Sharoni, 2016), while two used a mixed-method design (Dickson et al., 2013; Presseau et al., 2017), with the quantitative data from the latter being included in this review. Hammash et al. (2017) conducted a secondary analysis of baseline data obtained from a longitudinal prospective study on the factors contributing to the exacerbation of heart failure. The total number of participants across the studies was 2098, all of whom were adults aged 18 years or older. The sample sizes of the individual studies ranged from 30 to 609 participants. Six studies focused on adults with CHD (Presseau et al., 2017, included adults post-MI; Fang et al., 2017; Ginting et al., 2016; Presseau et al., 2017; Rocha et al., 2017; Rokhmah et al., 2020; Tawalbeh et al., 2015), while the remaining five studies included adults with heart failure (Dickson et al., 2013; Hammash et al., 2017; Khaledi et al., 2014; Mei et al., 2019; Yunus & Sharoni, 2016).

The Multidimensional Scale of Perceived Social Support (MSPSS, Zimet et al., 1988) was used to assess social support in five studies (Dickson et al., 2013; Ginting et al., 2016; Hammash et al., 2017; Khaledi et al., 2014; Mei et al., 2019). Fang et al. (2017) utilized the social support dimension of the social capital scale. Rocha et al. (2017) used the Instrumental-Expressive Social-Support Scale (IESSS) developed by Guerra (1995) to measure family support, while Yunus and Sharoni (2016) and Tawalbeh et al. (2015) used the Medical Outcomes Study (MOS) Social Support Survey (Sherbourne & Stewart, 1991) and the Personal Resource Questionnaire (PRQ, Weinert, 2000), respectively, to assess social support. Presseau et al. (2017) utilized a standardized questionnaire informed by the HAPA model to evaluate social support, which consisted of three items based on Molloy et al. (2010).

Self-care was measured using the Self-Care of Heart Failure Index (SCHFI Version 6.2, Riegel et al., 2009) in three studies (Dickson et al., 2013; Mei et al., 2019; Yunus & Sharoni, 2016), while Khaledi et al. (2014) used the European Heart Failure Self-care Behavior Scale (Jaarsma et al., 2003). The Adherence Scale (Arabic version) developed by Alm-Roijer et al. (2004) was used in Tawalbeh et al. (2015) to measure adherence to a healthy lifestyle. Hammash et al. (2017) and Ginting et al. (2016) used the Medical Outcomes Study (MOS)-specific Adherence Scale (DiMatteo et al., 1993) and Health Behavior Inventory (HBI), respectively, to measure adherence to treatment and health behaviors in individuals with CHD. The other three studies (Fang et al., 2017; Presseau et al., 2017; Rocha et al., 2017) measured physical activity, medication adherence, and smoking cessation using the Health Promoting Lifestyle Profile II (HPLPII, Walker et al., 1987), Morisky Medication Adherence Questionnaire (MMAS-8; Morisky et al., 2008), and treatment self-regulation questionnaire (Levesque et al., 2007), respectively. Rokhmah et al. (2020) was the only study that did not specify the questionnaires used to measure the variables.

In nine of the included studies, the relationship between the relevant variables was evaluated using Pearson’s correlation, while two studies used Spearman's rho (Dickson et al., 2013; Rokhmah et al., 2020). All the studies reported a significant positive correlation between the study variables (see Table 1). The correlation coefficients of all studies ranged from 0.10 to 0.75. Tawalbeh et al. (2015) found a strong positive correlation (*r* = 0.75) between social support and adherence to a healthy lifestyle, whereas Presseau et al. (2017) identified a weak correlation between social support and adherence to medication. Ginting et al. (2016) explored the relationship between social support and various health behaviors, including addictive behaviors, consumption of healthy and unhealthy foods, exercise, weight control, and medication adherence. While the authors provided a range of correlation values, they did not specify which values corresponded to each specific behavior.

**Table 1:** Characteristics of Included Studies (N = 11)

No.	Author and year	Country	Study Design	Population	Outcome
**1**	Fang et al. (2017)	China	Cross-sectional	609 adults with coronary heart disease	Social support was significantly positively associated with adherence to physical activity and nutrition respectively (*r* = 0.41, *r* = 0.48)
**2**	Rokhmah et al. (2020)	Indonesia	Cross-sectional	105 adults with coronary heart disease	Family support was significantly positively correlated with diet adherence (*r* = 0.626, *p* = .000)
**3**	Mei et al. (2019)	China	Cross-sectional	210 patients with heart failure	Men: Social support was significantly positively associated with self-care maintenance (*r =* 0.366, *p* < .01), self-care management (*r* = 0.418, *p* < .01) and self-care confidence (*r* = 0.458, *p* < .01) Women: Social support was significantly positively associated with self-care management (*r* = 0.262, *p* < .01) and self-care confidence (*r* = 0.248, *p* < .01), but not with self-care maintenance (*r* = 0.166)
**4**	Hammash et al. (2017)	USA	Cross-sectional	157 in-patients with heart failure	perceived social support was significantly positively correlated with adherence to treatment (*r* = 0.24; *p* = .003)
**5**	Rocha et al. (2017)	Portugal	Cross-sectional	110 patients hospitalized with acute coronary syndrome	Family social support was significantly positively associated with autonomous self-regulation for smoking cessation (*r* = 0.25, *p* < .05)
**6**	Ginting et al. (2016)	Indonesia	Cross-sectional	386 adults with coronary heart disease (CHD)	Most health behavior domains correlated significantly with perceived support from the family (*r* = −.19 to .16, *p* < .01), friends (*r* = −17 to .17, *p* < .05), and significant others (*r* = −.12 to .10, *p* < .05).
**7**	Yunus and Sharoni (2016)	Malaysia	Cross-sectional	113 adults with chronic heart failure (CHF)	Social support was significantly positively associated with self-care (*r* = 0.26, *p* < .05)
**8**	Tawalbeh et al. (2015)	Jordan	Cross-sectional	113 adults with coronary artery disease	Perceived social support was significantly positively correlated with adherence to a healthy lifestyle (*r* = .75; *p* ≤ .001)
**9**	Dickson et al. (2013)	USA	Mixed method	30 Black patients with HF	Social support was significantly positively correlated with self-care maintenance (*r* = 0.483, *p* = .008), self-care confidence (*r* = 0.384, *p* < .04) and not associated with self-care management (*r* = 0.204)
**10**	Khaledi et al. (2014)	Iran	Cross-sectional	64 adults with heart failure	Total perceived social support was significantly positively correlated with self-care behaviors (*r* = 0.481, *p* < .001). Family support had the strongest positive correlation with self-care behavior (*r* = 0.462, *p* < .001), while significant others and friends support had a significant positive correlation of *r* = 0.261, *p* < .001 and *r* = 0.331, *p* < .001 respectively
**11**	Presseau et al. (2017)	Canada (telephone interview)	Mixed method	201 adults post myocardial infarction	Social support was significantly positively associated with adherence to medication (*r* = 0.15, *p* < .05)

Therefore, the results from all of the studies indicate a significant positive relationship between social support and adherence to self-care behaviors in patients with CHD and heart failure. This indicates that higher levels of social support are associated with better adherence to self-care behaviors among patients with CHD and heart failure.

### Quality Appraisal

Among the 11 studies included in the analysis, ten studies clearly outlined the criteria for inclusion in the sample (Dickson et al., 2013; Fang et al., 2017; Ginting et al., 2016; Hammash et al., 2017; Khaledi et al., 2014; Mei et al., 2019; Presseau et al., 2017; Rocha et al., 2017; Tawalbeh et al., 2015; Yunus & Sharoni, 2016), while seven studies also provided criteria for exclusion (Dickson et al., 2013; Ginting et al., 2016; Hammash et al., 2017; Mei et al., 2019; Rocha et al., 2017; Tawalbeh et al., 2015; Yunus & Sharoni, 2016). The study subjects and settings are described in detail in all studies, except for Rokhmah et al. (2020). Fang et al. (2017) reported correlation values but did not provide *p*-values.

The variables were measured using reliable and valid measurements and appropriate statistical analyses in all studies, except for Rokhmah et al. (2020). Presseau et al. (2017) used three items based on Molloy et al. (2010) to measure social support, however, the internal consistency of these items was reported. The instruments used by Fang et al. (2017), Ginting et al. (2016), Mei et al. (2019), Rocha et al. (2017), and Tawalbeh et al. (2015) were translated into native languages (Chinese, Arabic, Portuguese) using expert back-to-back translations. Confounding factors were identified and strategies to address them were stated in the studies by Ginting et al. (2016), Hammash et al. (2017), Presseau et al. (2017), and Tawalbeh et al. (2015) and only two studies (Khaledi et al., 2014; Presseau et al., 2017) employed objective standard criteria to measure the condition and verify the diagnosis.

Eight of the selected studies failed to report the method for determining the sample size, while three studies (Presseau et al., 2017, Tawalbeh et al., 2015, Yunus & Sharoni, 2016) reported this information. Presseau et al. (2017) was the only study that met all quality appraisal criteria; however, it relied on telephonic interviews to collect data on HAPA predictors and medication adherence. This raises concerns about the extent to which psychological aspects and behavior are captured compared to in-person interviews, where the subject's non-verbal cues and behavior can be observed. These factors are particularly relevant when examining vulnerable populations, such as those with CVDs.

In addition to this, all the studies relied on self-report questionnaires, leading to a high likelihood of social desirability bias and recall bias. Most studies relied solely on subjective measures through self-reported questionnaires. Incorporating objective measures, such as drug monitoring and counting pills, alongside subjective measures would yield more reliable data. Furthermore, sample size calculation was not reported in most (eight) of the studies, indicating a potential methodological weakness. Additionally, one study that explored a model-based approach (Presseau et al., 2017) did not utilize a standardized instrument for measuring social support but used three items based on another study. This raises concerns regarding the reliability of the measurement and the generalizability of findings when using model-based approaches with limited tools available for certain factors of a model. Table 2 shows the quality appraisal checklist for each of the selected studies.

**Table 2:** Quality Appraisal Using the Joanna Briggs Institute Appraisal Checklist for Analytical and Cross-Sectional Studies

Quality criteria	Fang et al. (2017)	Rokhmah et al. (2020)	Mei et al. (2019)	Hammash et al. (2017)	Rocha et al. (2017)	Ginting et al. (2016)	Yunus and Sharoni (2016)	Tawalbeh et al. (2015)	Dickson et al. (2013)	Khaledi et al. (2014)	Presseau et al. (2017)
1. Were the criteria for inclusion in the sample clearly defined?	Y	N	Y	Y	Y	Y	Y	Y	Y	Y	Y
2. Were the study subjects and the setting described in detail?	Y	N	Y	Y	Y	Y	Y	Y	Y	Y	Y
3. Was the exposure measured validly and reliably?	Y	N	Y	Y	Y	Y	Y	Y	Y	Y	Y
4. Were objective, standard criteria used for measurement of the condition?	N	N	N	N	N	N	N	N	N	Y	Y
5. Were the confounding factors identified?	N	N	N	Y	N	Y	N	Y	N	N	Y
6. Were strategies stated to deal with the confounding factors?	N	N	N	Y	N	Y	N	Y	N	N	Y
7. Were the outcomes measured validly and reliably?	Y	N	Y	Y	Y	Y	Y	Y	Y	Y	Y
8. Was appropriate statistical analysis used?	Y	N	Y	Y	Y	Y	Y	Y	Y	Y	Y

*Note.* Y = Yes; N = No.

## Discussion

The present systematic review was conducted to identify and summarize studies investigating the relationship between social support and adherence to self-care behaviors in cardiovascular conditions such as CHD and heart failure. Adherence to self-care behaviors is crucial for individuals with chronic illnesses, and various psychological factors, including social support, have been recognized as influential. The findings indicated significant positive correlations between social support and adherence to self-care behaviors, suggesting that enhancing social support could play a vital role in promoting adherence among patients with CHD and heart failure (Liu et al., 2022; Park et al., 2021; Presseau et al., 2017; Saiwutthikul et al., 2021; Zhou et al., 2022).

The verbal encouragement and affective influence provided by family members and significant others can improve emotion regulation, decision-making, self-worth, and self-confidence, thereby enhancing self-care (Cohen & Wills, 1985, as cited in Hammash et al., 2017). Magrin et al. (2015) found that functional social support, rather than solely structural aspects such as living arrangements or marital status, significantly influence adherence to diet, physical activity, medication and smoking cessation among individuals with chronic conditions such as hypertension.

Khodaveisi et al. (2017) discussed the relevance of Pender’s Revised Health Promotion Model (HPM) in supporting the association between social support and medication adherence. According to the HPM, individuals are more likely to engage in healthy behaviors when they receive support, guidance, and encouragement from family, friends, and healthcare providers. These individuals serve as important sources of influence who can help improve the patient’s commitment to health-promoting behaviors (Tawalbeh et al., 2015). The Health Action Process Approach, which delineates the formation of health behavior in two phases (motivational and volitional), also highlights the significance of social support (Schwarzer, 2016). Social support is important in both phases, facilitating the translation of intention into behavior, however, its role becomes particularly crucial in the volitional phase. This is consistent with the finding that social support is significantly and positively associated with adherence to self-care behaviors.

The results highlight that only a few studies explored social support and adherence to self-care behavior as their primary aim (Khaledi et al., 2014; Tawalbeh et al., 2015; Yunus & Sharoni, 2016). For an in-depth understanding of the intricate relationship, it is essential to conduct empirical studies that focus solely on examining these constructs rather than incorporating them within broader models or frameworks.

### Limitations

The review has some limitations worth mentioning. CHD and heart failure were the only two conditions considered for this review as they involve significant lifestyle changes. In addition, relevant studies in other languages and databases had to be excluded because the selection of the studies was based on predefined criteria. Furthermore, all the studies were cross-sectional in design, preventing the establishment of a causal relationship between the study variables. Since only the *r*-value between the variables was considered, important information that other multivariate analyses could have provided was not considered. Finally, due to the limited number of studies and the heterogeneity of the methodology and instruments used, a meta-analysis could not be conducted.

### Strengths

The present systematic review followed the PRISMA guidelines and aimed to investigate the relationship between social support and adherence to self-care in individuals with coronary heart disease and heart failure based on recent literature. While some studies and reviews have focused on these variables in the context of heart failure alone, no previous systematic review has investigated CHD and heart failure together, despite the fact that CHD is a major cause of heart failure. This review also highlighted the importance of early psychological interventions in improving health outcomes and thereby reducing healthcare costs in chronic diseases such as cardiovascular diseases. The insights generated from the review can be effectively implemented in practice to improve self-care and mitigate the global burden of cardiovascular disease and fatalities.

### Implications

This systematic review found that most studies on self-care primarily focused on health-enhancing behaviors, such as diet and physical activity, with fewer studies on health-compromising behaviors, such as smoking and alcohol cessation. To gain a deeper understanding of the relationship between self-care and psychological factors, future research should consider both types of behaviors. Additionally, a more comprehensive understanding of self-care can be achieved through a theoretical model that considers all aspects of self-care adherence, such as the theory of self-care for chronic illnesses. By conceptualizing self-care through established theoretical models researchers can obtain a holistic perspective that can inform the development of interventions oriented along these theoretical foundations.

An important insight from this review is that studies on adherence and self-care mostly rely on self-reported measures, which may be influenced by bias. To enhance the accuracy of outcomes, future investigations should incorporate both self-reported and objective measurements, such as drug monitoring or electronic devices. Additionally, developing scales with robust psychometric properties, and combining them with other objective measures, could provide a clearer understanding of self-care behaviors in future research and practice.

Adequate self-care involves the development of tactical and educational skills over time with the involvement of family and friends. Traditional patient education may be insufficient, and interventions involving trusted resources are necessary to encourage self-care and rectify misconceptions. Culturally sensitive community-based interventions are needed to reduce social isolation and provide social support resources for individuals with chronic illnesses and their families. Existing self-care interventions are mostly patient-centered and neglect the influence of family members, which is detrimental to improving self-care.

The construct of social capital, which includes social support, social participation, and social trust, is beneficial in improving adherence to self-care. Personality is also an important factor in how social support is perceived and utilized by individuals in self-care. Therefore, interventions should consider the multidimensional nature of social support and tailor it to individual needs, including personality traits and specific types of social support.

Self-care is a complex process that requires careful consideration. When addressing social support in the context of self-care for cardiovascular conditions like CHD and heart failure, all relevant factors must be taken into account. Tailored interventions that focus on an individual's unique genetic makeup, sociodemographic factors, available resources, and illness severity are more effective than generic interventions targeting all patients.

### Conclusion

This systematic review provides a comprehensive overview of the evidence linking social support and adherence to self-care behaviors in patients with CHD and heart failure. The review highlights the need to develop rigorous standardized tools for measuring situational and context-specific variables related to adherence to self-care behaviors. Additionally, the findings underscore the significance of conducting studies with robust methodological design and designing interventions that are grounded in appropriate theoretical framework, personality factors and socio-cultural influences.

## Supplementary Materials

For this article, the complete search string used for the study is available (see Babygeetha & Devineni, 2023).



BabygeethaA.
DevineniD.
 (2023). Supplementary materials for: Social support and adherence to self-care behavior among patients with coronary heart disease and heart failure: A systematic review
[Search string]. PsychOpen. 10.23668/psycharchives.13158
PMC1093666338487598
